# Induction of Apoptosis in Colorectal Cancer Cells by Zinc Oxide Nanoparticles Extracted From *Lactiplantibacillus plantarum* With Gene Expression Control of Apoptosis‐Related Genes

**DOI:** 10.1155/bmri/4464604

**Published:** 2026-04-15

**Authors:** Mohammadreza Azimi, Anoosh Eghdami, Bahram Keyvani, Ramin Cheraghali

**Affiliations:** ^1^ Department of Biochemistry, Medical Faculty, Saveh Branch, Islamic Azad University, Saveh, Iran, azad.ac.ir

**Keywords:** apoptosis, HK2, *Lactiplantibacillus plantarum*, nanoparticle, pkm2, zinc oxide

## Abstract

Colorectal cancer is the third leading cause of cancer death worldwide. Zinc oxide nanoparticles (ZnO NPs) have garnered attention as potential anticancer agents in cancer therapy research. Synthesis of metal nanoparticles using beneficial bacteria is considered one of the most effective methods for cancer treatment, with minimal side effects. This study is aimed at synthesizing ZnO nanoparticles from *Lactiplantibacillus plantarum* biofilm and investigate their impact on cell division and apoptosis‐related gene expression in colorectal epithelial cancer cells (HT‐29) under laboratory conditions. After isolating and validating ZnO nanoparticles in the *L. plantarum* biofilm, HT‐29 cells were cultured with different concentrations of zinc oxide nanoparticles for 24 h, and MTT was used to determine IC_50_. Subsequently, the studied cells were cultured with 5 and 10 *μ*g of ZnO NPs for 24 h, and Western blot, real‐time PCR, and Annexin V‐PI staining were used to evaluate the role of ZnO NPs in inducing apoptosis and cell division in HT‐29 cells. The MTT assay results revealed that a 40 *μ*g/L concentration showed the highest cytotoxicity after 24 h. It was also found that cell viability cultured with ZnO NPs was downregulated in a dose‐dependent manner. Additionally, ZnO NPs upregulated the expression of BAX, caspase 3, and 9 genes while downregulating the expression of Bcl‐2, PKM2, and HK2 genes. The current study demonstrated the potential of the *L. plantarum* biofilm to produce ZnO nanoparticles. Moreover, ZnO NPs induced apoptosis and downregulated cell division‐related genes in HT‐29 cells. These findings provide a foundation for further research and the utilization of zinc oxide nanoparticles in the pharmaceutical and colorectal cancer therapy industries.

## 1. Introduction

Colorectal cancer is a prevalent form of cancer worldwide, resulting in many deaths annually [1, 2]. Genetic and epigenetic alterations that transform the normal colonic epithelium into colorectal cancer [3, 4] contribute to the development of this cancer. Several treatment options are currently available for cancer, including surgery, chemotherapy, radiation therapy, and palliative care. These treatments are combined, depending on the location and stage of the cancer [5, 6]. However, these treatments often impact healthy cells, leading to patient side effects [7, 8]. Identifying novel cancer treatment methods is a critical global public health concern [6–9]. In this regard, nanotechnology has provided valuable tools for discovering and developing innovative cancer therapies. One significant advancement is the use of modified nanoparticles for targeted, selective drug delivery to cancerous tissues. Among these nanoparticles, zinc oxide nanoparticles (ZnO NPs) have been recognized as safe substances by the US Food and Drug Administration, making them attractive candidates for drug therapy [7, 10–13].

The application of ZnO and other metal nanoparticles in biomedical and oncological contexts has become increasingly significant due to their unique chemical and physical properties. Their size, comparable with biological molecules, enables them to penetrate cells and interact with biological systems. Recent investigations have demonstrated the anticancer efficacy of ZnO nanoparticles, positioning them as promising candidates for cancer therapy [11, 13].

.Although various methods for synthesizing nanoparticles exist, biocompatible approaches using bacteria, fungi, and plants offer simple and cost‐effective alternatives to chemical and physical methods [14, 15]. Numerous microorganisms, including bacteria, fungi, and yeasts, have been investigated for their efficiency in zinc ion (Zn^2+^) uptake and zinc oxide (ZnO) nanoparticle synthesis. For instance, bacteria like *Lactobacillus* can produce intracellular ZnO nanoparticles [16, 17], whereas fungi and yeasts, such as *Aspergillus aeneus* and *Pichia fermentans*, can produce extracellular ZnO nanoparticles [18, 19].

Lactic acid bacteria (LAB) have garnered significant attention among microorganisms due to their safety in transportation, food‐grade status, and potential probiotic properties, which are beneficial for both human and animal consumption and contribute to health promotion [20, 21]. Moreover, in several studies, LAB has demonstrated efficiency in mediating the synthesis of ZnO NPs [22, 23]. Previous studies have reported that LAB can produce gold nanoparticles [24], palladium nanoparticles [25], and ZnO NPs [26], thus making it a valuable cellular factory for nanoparticle synthesis. With a thick cell wall consisting of peptidoglycan, lipoteichoic acid, proteins, and polysaccharides, LAB serves as a site for the adsorption and bioreduction of metal ions due to its negative electrokinetic potential, which attracts metal cations and initiates the biosynthesis of nanoparticles [27, 28].

*Lactiplantibacillus plantarum* was selected for the biosynthesis of ZnO NPs due to its well‐documented safety profile, food‐grade status, and probiotic characteristics, making it an ideal candidate for biomedical applications. The strong cell wall of the bacterium, which consists of peptidoglycan, lipoteichoic acid, protein, and polysaccharides, generates a negative electrokinetic potential that facilitates the adsorption and bioreduction of zinc ions (Zn^2+^), leading to the effective synthesis of nanoparticles. Additionally, *L. plantarum* has been reported to be robust and capable of synthesizing multiple metal nanoparticles, such as ZnO NPs, as supported by previous studies [29]. Its use offers an inexpensive, green, and biocompatible alternative to chemical and physical synthesis methods, aligning with the aim of this research to develop green nanotechnology for cancer therapy.

Notably, *Lactobacillus plantarum* is particularly resistant and is a vital natural source of ZnO NPs [29]. Therefore, considering the significance of nanoparticle‐based cancer treatment, the present study is aimed at producing ZnO NPs using *Lactobacillus plantarum* bacteria and induce apoptosis in (HT‐29) cancer cells.

## 2. Materials and Methods

### 2.1. Biosynthesis of ZnO NPs

We obtained lyophilized powder of *L. plantarum* from the Iranian Research and Industrial Scientific Center (Bacterial and Fungal Collection) for the synthesis of ZnO NPs. We added a specific amount of the powder to a sterile liquid growth medium and incubated it at 30°C for 18 h to facilitate logarithmic growth.

We inoculated *L. plantarum* into a 750 mL Erlenmeyer flask containing 100 mL of De Man, Rogosa, and Sharpe (MRS) broth to conduct the biosynthesis study. We incubated the flask at 37°C with agitation at 120 rpm for 24 h. After incubation, we centrifuged the culture at 2800 × g for 10 min, collecting the cell biomass and supernatant separately.

To summarize, the biomass was washed three times with sterile phosphate‐buffered saline (PBS) before being resuspended in 50 mL of sterile deionized water containing 500 mM Zn^2+^. We subsequently incubated the suspension at 37°C with agitation at 120 rpm for 24 h to initiate biosynthesis. Afterward, we collected the biomass by centrifugation and disrupted the cells using ultrasound at 30°C for 30 min to obtain biosynthesized ZnO NPs. We purified the biosynthesized ZnO NPs using high‐speed centrifugation (18000 × g for 30 min).

### 2.2. MTT Assay

MTT is a yellow‐colored tetrazolium salt that is soluble in water. It undergoes reduction by dehydrogenases in the active mitochondria of living cells, forming insoluble formazan crystals. These crystals appear as purple‐colored deposits. The intensity of the purple color is directly proportional to cellular activity and the number of viable cells. Approximately 5 × 10^4^ cells (counted using Trypan Blue staining) were seeded in a 96‐well culture plate for this assay. Subsequently, we exposed them to various concentrations of ZnO NPs (5, 10, 20, and 40 *μ*g/mL) for 24 h. After the specified time intervals, we added 20 *μ*L of a 5‐mg/mL MTT solution to each well. We then incubated the plate for 3 hours at 37°C in a CO_2_ incubator. Next, we added 100 *μ*L of dimethyl sulfoxide (DMSO) (Merck, Germany) to each well. Finally, we measured the plate′s optical absorbance at 570 nm using a spectrophotometer after a 15‐min incubation at room temperature.

To ensure reliable, accurate results, we repeated each experiment five times. We calculated the percentage of viable cells using the following formula [5]:

100 × Mean absorbance of control sample/mean absorbance of treated sample = percentage of viable cells.

### 2.3. Cell Counting

After culturing and initially counting HT‐29 cells, we transferred 3 × 10^5^ HT‐29 cells per milliliter into six‐well plates. We divided them into two groups: Group 1 treated HT‐29 cells with 5 *μ*g/mL of ZnO NPs (incubated for 24 and 48 h), and Group 2 comprised untreated HT‐29 cells. We then performed cell counting and analyzed cell morphology using light and electron microscopy.

### 2.4. Flow Cytometry

To determine the percentage of apoptotic cells in HT‐29 cells treated with v (5 *μ*g/mL), flow cytometry was performed using the Annexin V and PI method, following the protocol. HT‐29 colon cancer cells were treated with ZnO NPs for 24 and 48 h, whereas the untreated cells served as the control group. Finally, we examined the percentage of cells undergoing initial apoptosis, apoptosis, and necrosis.

We analyzed the data using FLOW J V10 and GraphPad Version 8 software programs. The chart presents the results divided into four quadrants: Q1 represents healthy cells (cells not stained with Annexin V and PI), Q2 represents cells in early apoptosis (cells stained only with Annexin V), Q3 represents cells in late apoptosis (cells stained with Annexin V and PI), and Q4 represents cells undergoing necrosis (cells stained only with PI).

### 2.5. Lactate Dehydrogenase (LDH) Test

We cultured cells in 24‐well plates with a specific concentration of ZnO NPs (5 *μ*g/mL) for 24 and 48 h at 37°C. Afterward, we collected and lysed the cells. We measured LDH activity in both the supernatant and cell lysate using the LDH detection kit from Pars Azmoon, Iran.

### 2.6. RNA Extraction and cDNA Synthesis

We extracted total RNA from cells using the DNaseasy Kit (Asia). After analyzing the extracted RNA using NanoDrop and an agarose gel, we converted it to cDNA using the OneTaq Kit (Azuma). Subsequently, we separated the cDNA strands at 95°Cfor 10 min, based on the annealing temperature determined by the formula. Following this, we conducted a 40‐cycle process for PCR amplification and quality analysis of the primers on a 2% agarose gel. This process consisted of 10 s at 95°C, 20 s at 60°C, and 20 s at 72°C.

### 2.7. Primer Design and Quality Analysis by Agarose Gel Electrophoresis

We acquired the exon sequences from the NCBI and Ensembl databases in the current study. Next, we designed primers using the Beacon Designer software, targeting either the forward or reverse exon or the junction between two exons. We examined the primers for position, hairpin, and different bands to ensure their quality using Beacon and Oligo software, as well as the NCBI database. Once we ordered and received the primers, we diluted them appropriately and followed the manufacturer′s protocol (Table 1).

**Table 1:** Different RT‐qPCR primers used in the study.

Gene (ENST)	Sequence (5^′^—3^′^)	T_m_ (°C)	Length (bp)
Caspase 3(ENST00000393585.6)	F: ATGGGAGCAAGTCAGTGGACR: CGTACCAGAGCGAGATGACA	60	84
Caspase 9(ENST00000469637.1)	F: GGCGGAGCTCATGATGTCTGTGR: TTCCGGTGTGCCATCTCCATCA	61	156
BCL‐2(ENSG00000126453)	F: GAGCGTCAACAGGGAGAR: GCCAGGAGAAATCAAACA	60	164
Bax(ENSG00000087088)	F: ACTAAAGTGCCCGAGCTGAR: ACTCCAGCCACAAAGATGGT	60	161
HK2‐201 (ENST00000290573.7)	F: TGGCTAACTTCATGGATAAR: CAGGAAACTCTCGTCTAG	57	104
PKM‐202(ENST00000335181.10)	F: GAGACGTTGAAGGAGATGAR: CGCACATTCTTGATGGTC	60	101
B‐ACTIN(ENST00000515712.1)	F: CTACCTTCAACTCCATCAR: GAGCAATGATCTTGATCTTC	60	165

### 2.8. Real‐Time Analysis

We analyzed all biological samples (in groups of five replicates) using the Rotor‐Gene Q 2.3.5 real‐time instrument manufactured by Rotor‐Gene. We followed a thermal protocol, starting at 95°C, to separate cDNA strands for 10 min. The cycling program included 40 cycles for amplification and reading, with the following temperature settings: 95°C for 10 s, 60°C for 20 s, and 72°C for 20 s. For the experiment, we utilized the Master Mix kit and SYBR Green from Yekta TajhizAzma. Initially, we performed cDNA normalization using a control curve. Subsequently, we obtained the melting curve and CT values for each analyzed sample.

We conducted the initial analysis using GeneX v6.7 software and reported all results as delta–delta CT and log2. Afterward, we performed statistical analysis using GraphPad Prism Version 8 software.

### 2.9. Western Blotting

To determine protein expression levels, we performed a Western blot analysis. Cells were lysed with 70 *μ*L of Phosphosafe after 24 h of treatment with ZnO NPs (5 *μ*g/mL). The protein concentration (20 *μ*g) was then determined using the BCA protein assay. We performed electrophoresis using a Nu‐PAGE 10% SDS‐PAGE Bis‐Tris gel in an SDS‐PAGE buffer. Then, we transferred the samples onto a polyvinylidene fluoride (PVDF) membrane. We blocked the membrane with 3% bovine serum albumin and washed it with Tris‐buffered saline containing Tween 20 (TBST). The membrane was then incubated overnight with primary antibodies (procaspase‐3, procaspase‐8, procaspase‐9, Bcl‐2, BAX, and *β*‐actin) diluted 1:1000. The membrane was then washed three times with TBST, and a secondary antibody (1:1000) was added. After incubation for 1 h, the membrane was rewashed with TBST. We detected band intensity using the Supersignal Femto chemiluminescent substrate kit and analyzed band density using ImageJ V1.46a software (Bethesda, Maryland, United States) [6].

### 2.10. Genotoxic Effects

To assess the effects of ZnO NPs on HT‐29 cells following 24 and 48 h of treatment, we conducted DNA extraction using the Yekta test kit. The cells were exposed to 5 *μ*g/mL ZnO NPs, whereas the untreated cells served as the control group. Subsequently, the extracted DNA was analyzed by gel electrophoresis.

### 2.11. BrdU Assay

We measured the inhibitory effect of ZnO NPs on HT‐29 cell proliferation by quantifying the incorporation of bromodeoxyuridine (BrdU) into the DNA of HT‐29 cells. To perform this analysis, we used a cell proliferation ELISA kit (colorimetric method) according to the manufacturer′s instructions. An ELISA reader (at a wavelength of 405 nm) was used to measure the optical absorption of the samples [6].

## 3. Results

We added the synthesized nanoparticles to deionized water and recorded the absorption peak at 360 nm for ZnO NPs (Figures 1a, 1b, 1c, and 1d). Previous studies have shown that ZnO NPs demonstrate an absorption peak between 340 and 380 nm, indicating surface plasmon resonance (SPR).

Figure 1(a) Scanning electron microscopy (SEM), (b) transmission electron microscopy (TEM), (c) Nanoparticle synthesis diagram. Figure (a) shows nanoparticle growth SEM at a magnification of 100,000×, illustrating size magnification. Figure (b) displays the TEM at a magnification of 200,000×. Figure (c) represents the chemical structure of the synthesized nanoparticle and its x‐ray diffraction pattern. (d) UV–visible spectrum of zinc oxide nanoparticles synthesized by *Lactiplantibacillus plantarum*. Note. Abs = absorbance.

**Figure:**
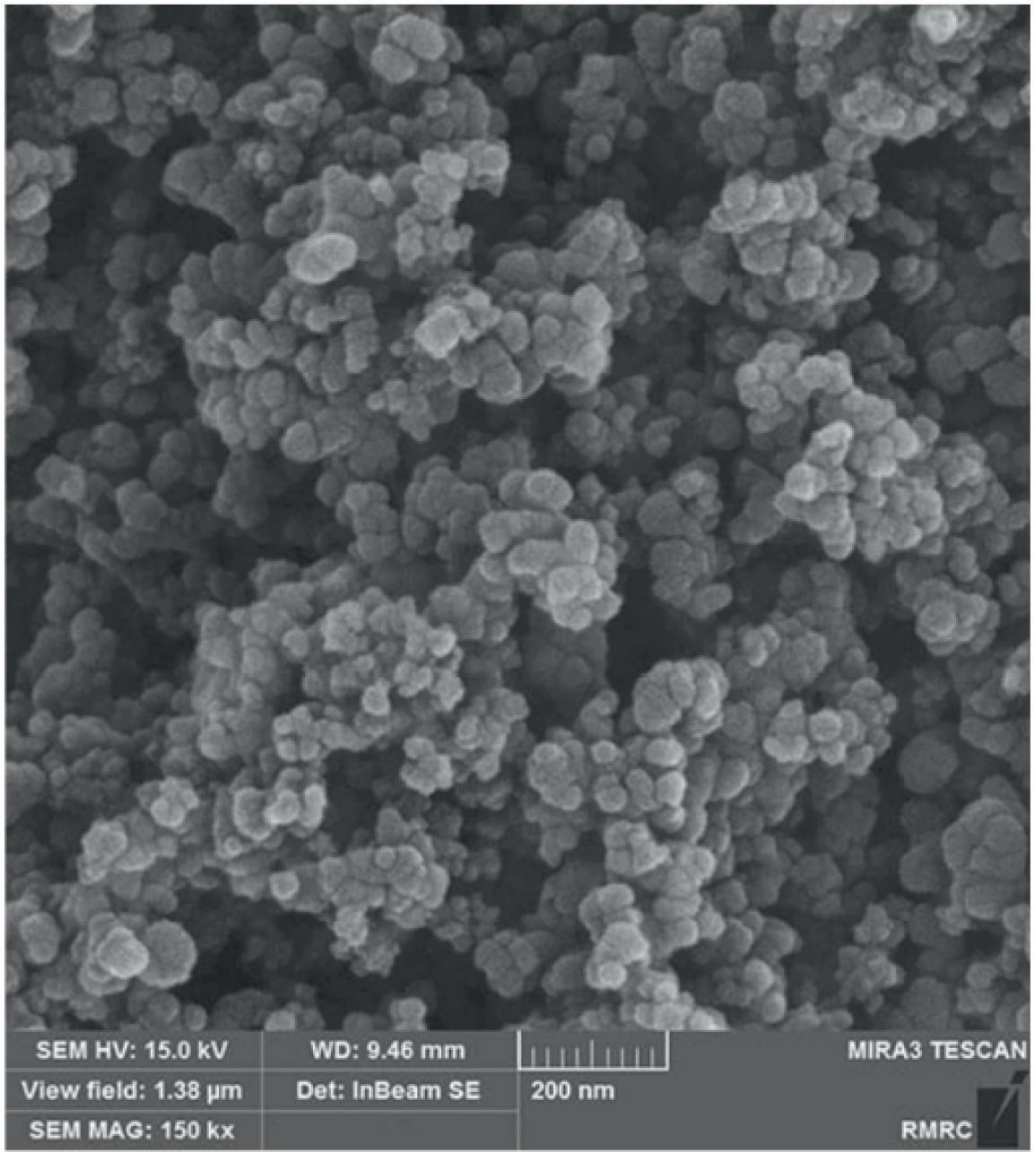
(a)

**Figure:**
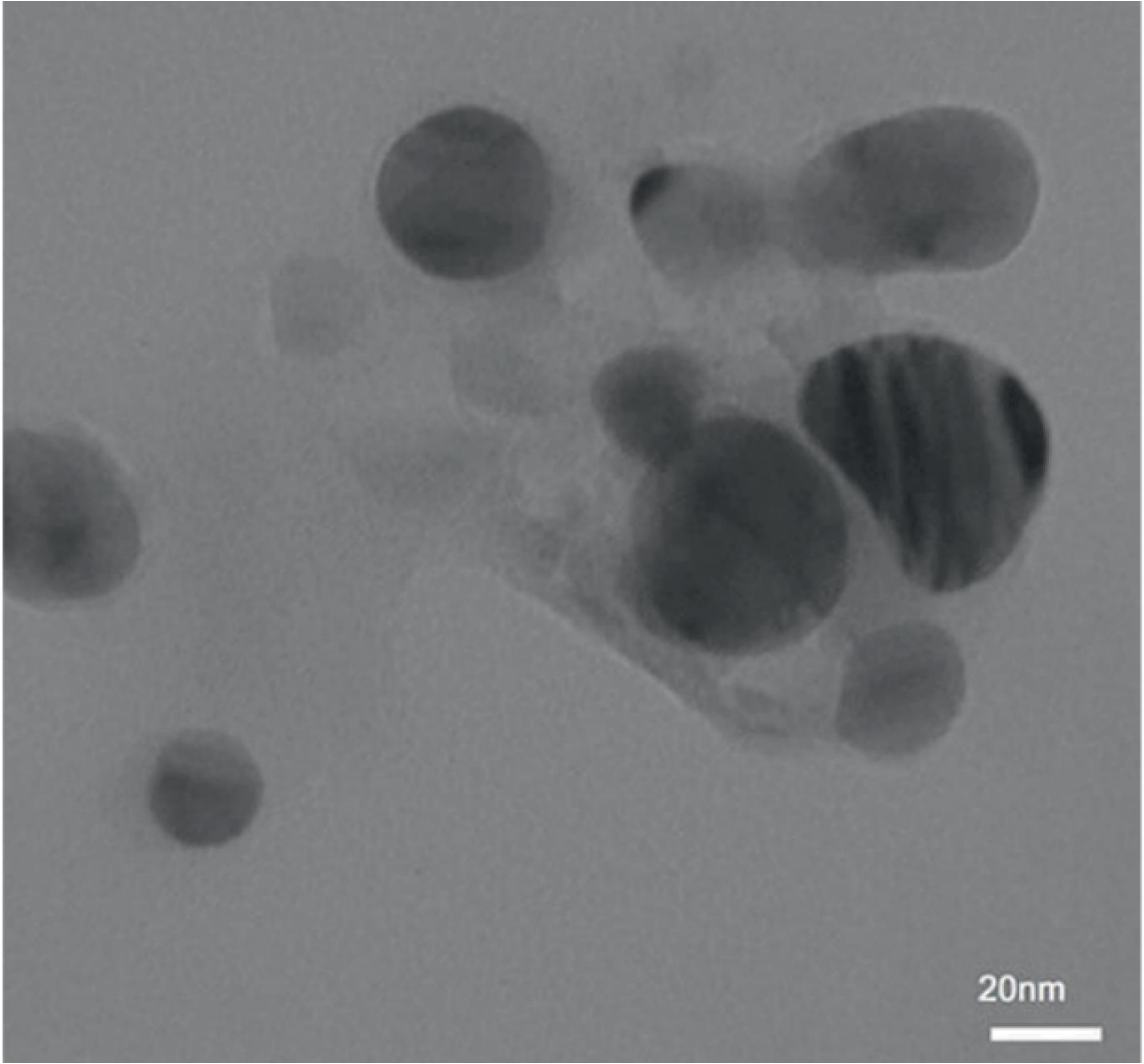
(b)

**Figure:**
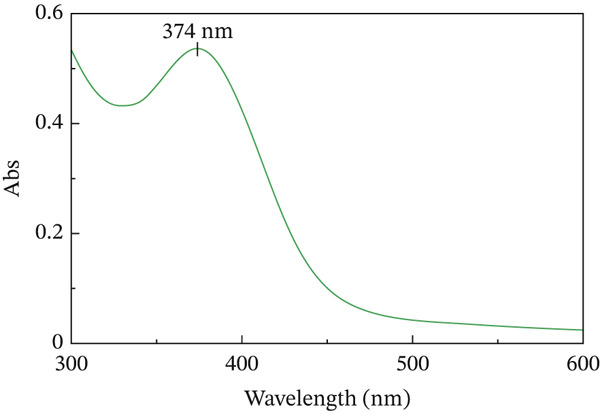
(c)

**Figure:**
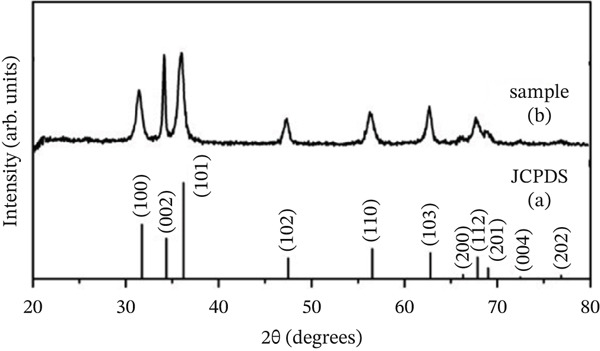
(d)

### 3.1. Anticancer Studies

#### 3.1.1. Anticancer Activity of ZnO NPs

**Figure 2:**
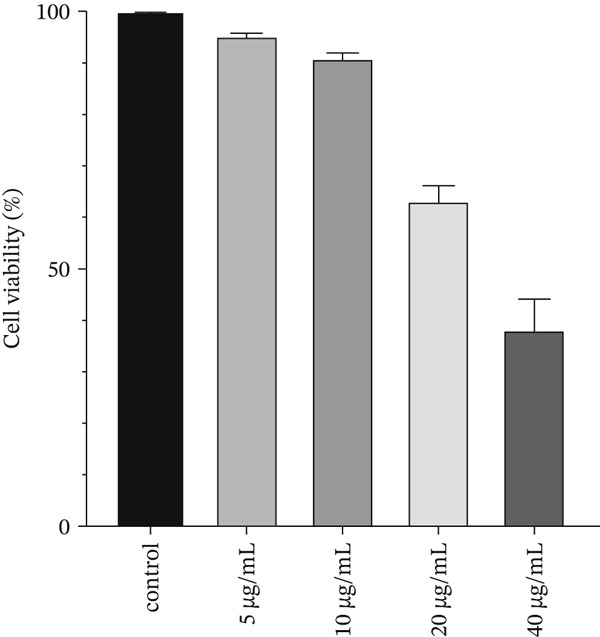
Depicts the highest cytotoxicity results observed at 40 *μ*g/mL concentration in the MTT assay.

We investigated the toxicity of ZnO NPs using the MTT method and observed a direct correlation between the concentration of ZnO NPs and cellular toxicity. We exposed HT‐29 cells to ZnO NPs for 24 h and determined the IC_50_ to be 40 *μ*g/mL (Figure 2). To analyze the impact of ZnO NPs on gene expression related to cell division and apoptosis, we selected concentrations of 5 and 10 *μ*g/mL based on the MTT assay results.

#### 3.1.2. Apoptosis Following (ZnO NPs) Treatment

Flow cytometry utilizing Annexin V and PI staining was employed to assess the induction of apoptosis in colon cells (HT‐29) treated with ZnO nanoparticles. Incubation of HT‐29 cells with 5 and 10 *μ*g/mL of ZnO NPs for 24 h resulted in concentration‐dependent apoptosis. Figures 3a, 3b, 3c, and 3d displays the flow cytometry results, indicating significant apoptosis in the cells treated with ZnO NPs. The percentage of cells undergoing apoptosis in the groups treated with 5 and 10 *μ*g/mL of ZnO NPs was 13.1*%* ± 0.52*%* and 21.2*%* ± 0.69*%*, respectively (Figures 3b, 3c, and 3d).

Figure 3(a–c) Figures related to Annexin V and PI flow cytometry. (Q1: Cells undergoing necrosis, Q2: Cells undergoing secondary apoptosis, Q3: cells undergoing apoptotic cell death, Q4: healthy cells). (d) Represents the average graph of cells undergoing apoptosis in different groups.

**Figure:**
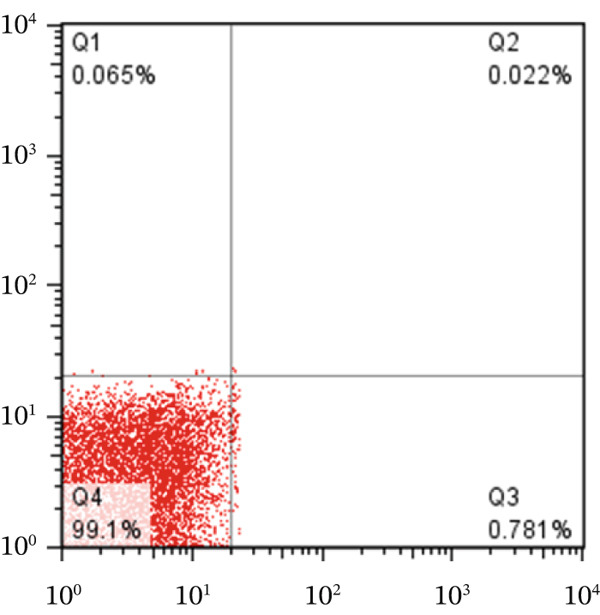
(a)

**Figure:**
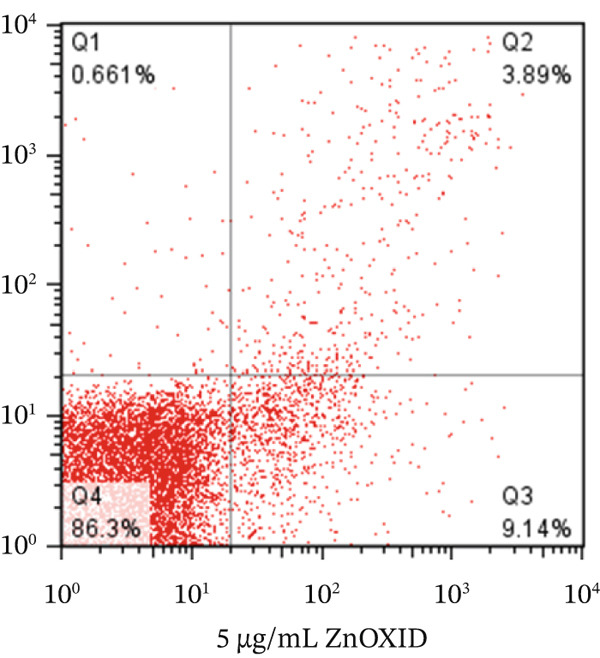
(b)

**Figure:**
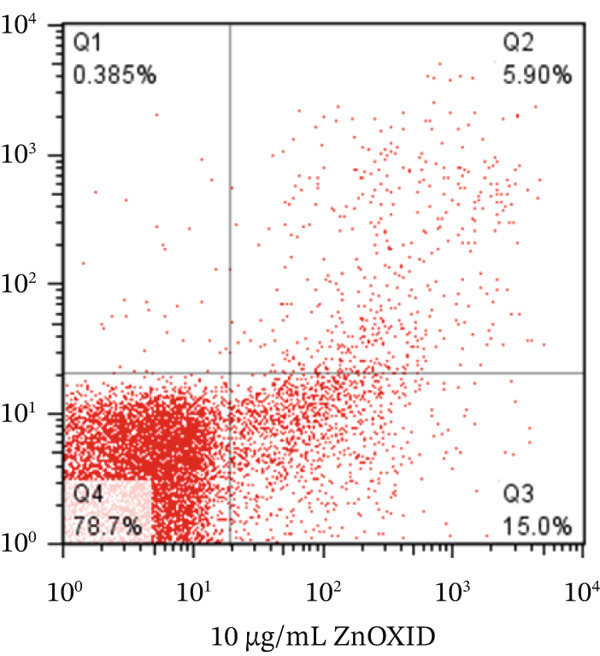
(c)

**Figure:**
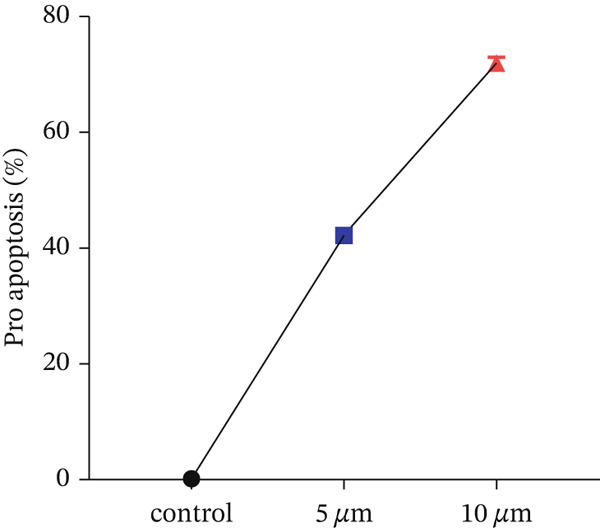
(d)

#### 3.1.3. Effect of ZnO NPs on the Expression of Genes Related to Apoptosis

**Figure 4:**
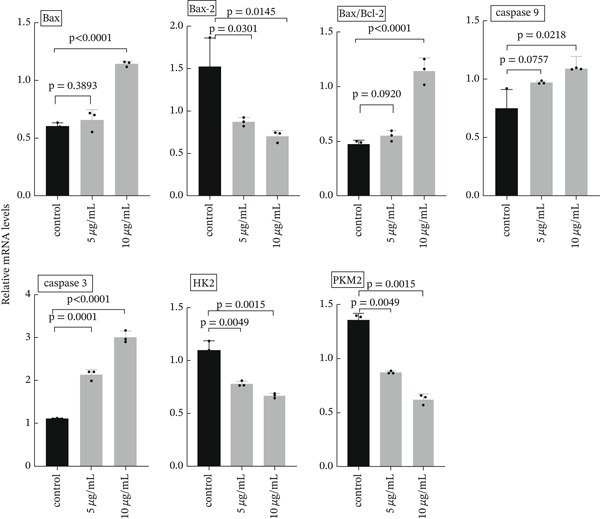
The expression of caspase‐3 and caspase‐9, Bcl‐2, BAX, and PKM2 genes at the mRNA level in (HT‐29) cancer cells was evaluated after 24 h of incubation with concentrations of 5 *μ*g/mL and 10 *μ*g/mL of ZnO NP. A comparison of fold change values for the expression of caspase‐3 and caspase‐8 genes was performed between ZnO NP‐treated and untreated cells, using internal controls of *β*‐actin.

**Figure 5:**
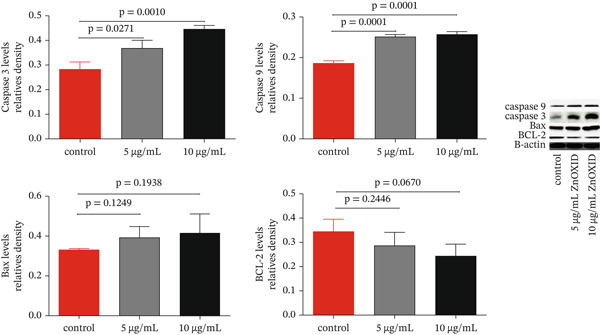
Expression of caspase‐3 and caspase‐9 genes, Bcl‐2, BAX, and PKM2 proteins in HT‐29 cancer cells after 24 h of incubation with 5 and 10 ZnO NP concentrations.

The expression levels of caspase 3 and 9 genes, as well as the BAX gene, at the mRNA and protein levels, increased in a dose‐dependent manner in the recipient groups exposed to 5 and 10 *μ*g/mL of ZnO NPs, compared with the control group (Figures 4 and 5). However, the expression level of the Bcl‐2 gene decreased in a dose‐dependent manner in the recipient groups exposed to 5 and 10 *μ*g/mL of ZnO NPs, compared with the control group (Figures 4 and 5).

#### 3.1.4. The Impact of ZnO NPs on the Expression of Cell Division‐Related Genes

The mRNA expression levels of PKM2 and HK2 genes showed a dose‐dependent decrease in the recipient groups exposed to 5 and 10 *μ*g/mL of ZnO NPs, compared with the control group (Figure 4).

#### 3.1.5. Morphological Changes and Accumulation of (ZnO NPs) Inside Cells

**Figure 6:**
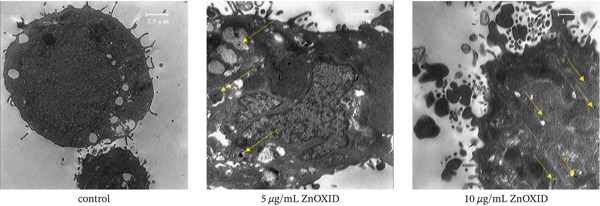
Transmission electron microscopy (TEM) demonstrating the dose‐dependent effects of ZnO nanoparticles (NPs) on apoptosis, cellular changes, and intracellular disarray in HT‐29 cells. The yellow arrows highlight the accumulation of ZnO NPs.

TEM electron microscopy was used to investigate intracellular changes and identify the site of nanoparticle accumulation. The findings revealed that cells treated with ZnO NPs exhibited cell‐surface wrinkling and early signs of apoptosis. In the control group cells, nuclear chromatin and intact organelles such as the endoplasmic reticulum, mitochondria, and ribosomes were observed (Figure 6). In contrast, chromatin in ZnO NP‐treated cells appeared dispersed and accumulated in various parts of the cell, whereas the endoplasmic reticulum, mitochondria, and ribosomes exhibited disruptions. Moreover, the nanoparticle accumulation site was determined to be within the cytoplasm and various organelles, including endosomes.

#### 3.1.6. Investigation of the Effect of ZnO NPs on DNA

The study employed agarose gel electrophoresis to assess DNA fragmentation induced by ZnO NPs in HT‐29 cells. Compared with the control group, an additional flame‐like band in the ZnO NPs‐treated groups indicates DNA damage (Figure 7a).

Figure 7Figure (a) depicts DNA in an agarose gel, showing an additional band indicating DNA fragmentation at different concentrations of ZnO NPs. Figure (b) represents the average results of DNA synthesis.

**Figure:**
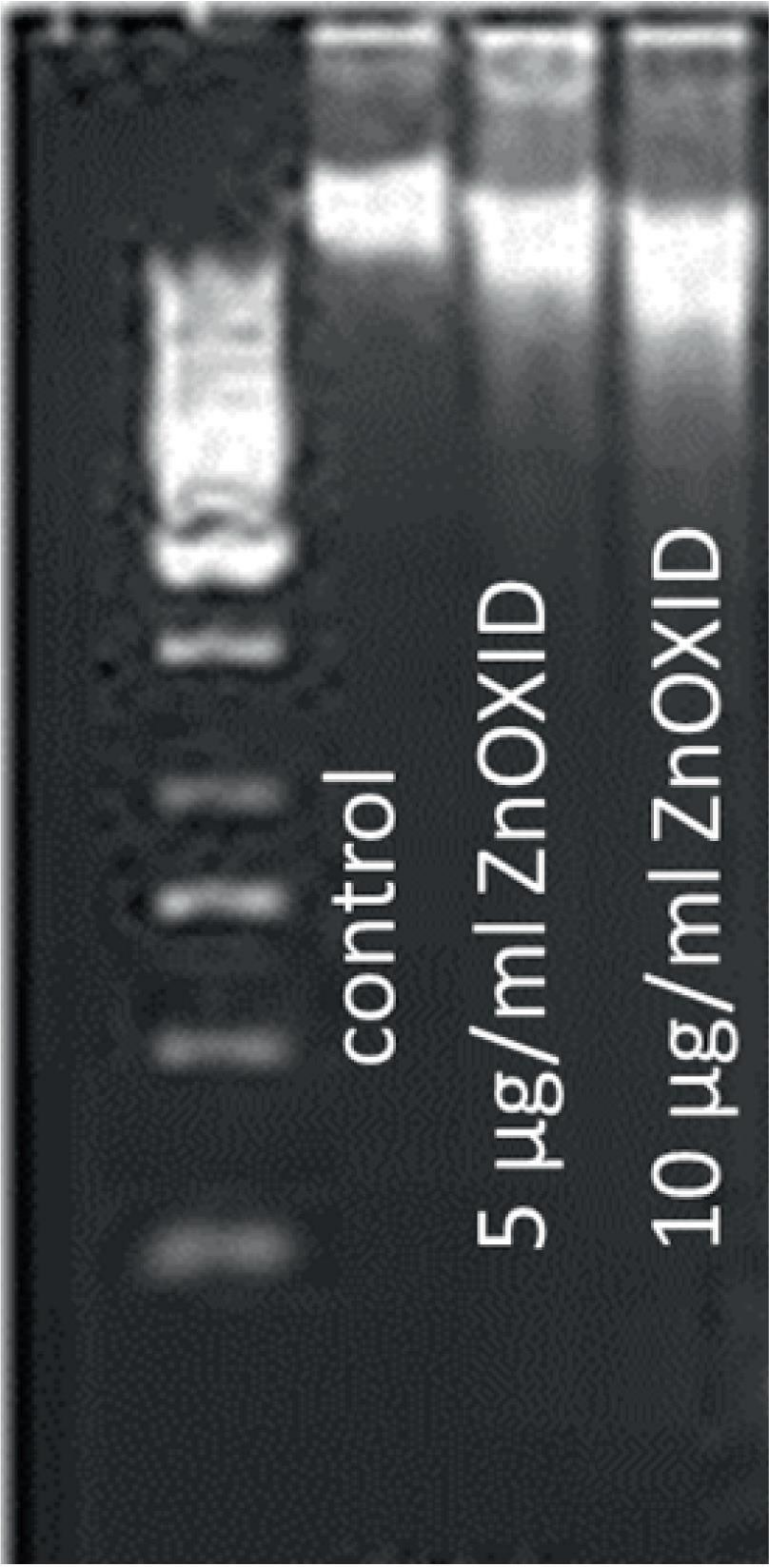
(a)

**Figure:**
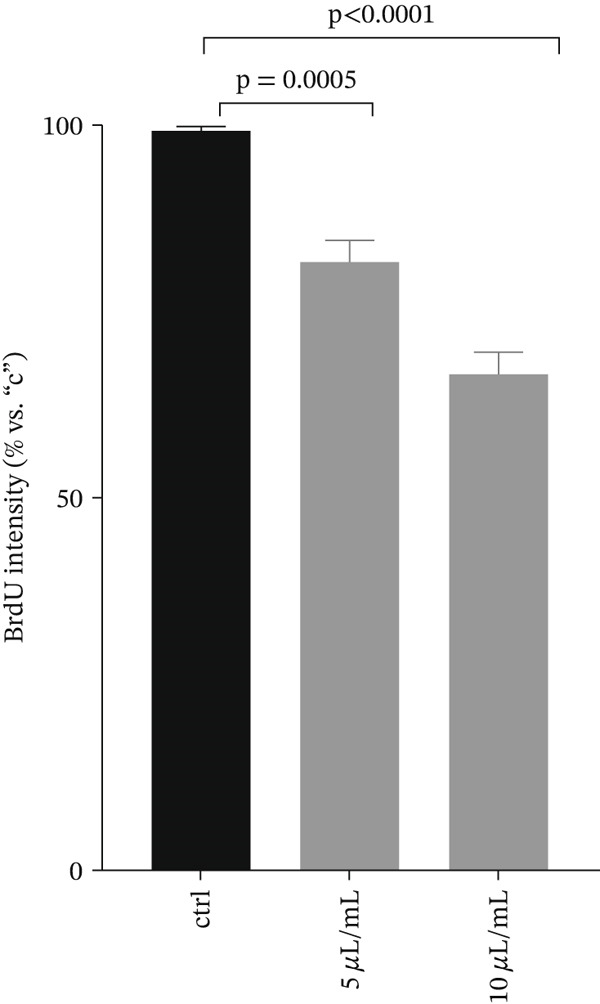
(b)

The BrdU method was utilized to quantify DNA synthesis. ZnO NPs inhibited DNA synthesis and arrested the cell cycle in a dose‐dependent manner (Figure 7b).

## 4. Discussion

ZnO NPs are versatile metal oxide nanoparticles with various biological applications, such as antibacterial properties and protection against ultraviolet radiation [30–32]. Recently, ZnO NPs have garnered significant attention in tumor therapy [32, 33]. In this study, we initially extracted ZnO NPs from the biofilm of *Lactobacillus plantarum* and examined their effects on the expression of genes associated with cell division and apoptosis in epithelial colorectal cancer cells. The results, obtained through MTT assay, flow cytometry, and transmission electron microscopy (TEM), indicate that ZnO NPs induce cell death and apoptosis in HT‐29 cells in a dose‐dependent manner.

*L*.*p* *l* *a* *n* *t* *a* *r* *u* *m*, as a nontoxic and naturally occurring probiotic, enables a green synthesis procedure that is biocompatible and environmentally friendly. It reduces environmental pollution and energy consumption because it occurs under mild conditions (e.g., 37°C in an aqueous medium). Highlight that nanoparticle synthesis using microbes is green, cost‐effective, and scalable, enabling sustainability in nanomedicine production. The use of renewable biological resources, such as *L. plantarum*, facilitates a circular economy by minimizing waste and utilizing biodegradable materials, with applications in the scalable and sustainable production of ZnO NPs for biomedical purposes [34, 35].

Recent studies have demonstrated that ZnO NPs exhibit antitumor activity through various mechanisms, including the induction of reactive oxygen species (ROS) and the modulation of apoptosis‐related gene expression [33]. The induction of ROS is considered one of the mechanisms by which ZnO NPs cause cancer cell death [36, 37]. Elevated levels of ROS lead to a decrease in the function of antioxidant enzymes in cells, ultimately resulting in oxidative damage to cells and tissues [38]. Studies have shown that ROS regulates the translocation, phosphorylation, and cleavage of proapoptotic Bcl‐2 family members, including Bax, Bak, Bad, Bid, Bim, and others, thereby inducing apoptosis [39, 40].

Previous studies have demonstrated that ZnO nanoparticles (NPs) can induce apoptosis and cell death in cancer cells. For example, DingPing Bai (2017) reported that ZnO nanoparticles triggered apoptosis and autophagy in breast cancer cells by damaging DNA and modulating the expression of genes related to apoptosis [41]. These findings are consistent with the results of the present study. Furthermore, Selvakumari et al. (2015) found that a concentration of 30 *μ*g/mL of ZnO nanoparticles resulted in the death of 50% of MCF‐7 breast cancer cells, supporting our findings [17].

TEM results revealed the accumulation of ZnO NPs within the cell and its organelles, including endosomes. These findings confirm the results of studies by Gilbert et al. (2009) and Lai et al. (2015). Furthermore, these studies demonstrated that the accumulation of nanoparticles on the cell surface and within organelles may alter their size [42, 43].

Multiple studies have shown that ZnO NPs upregulate caspases [36, 38], leading to cytotoxicity in cancer cells. In our study, our data clearly showed that caspase 3 and 9 expression increased in a dose‐dependent manner upon exposure to ZnO NPs. Numerous studies have corroborated these findings and have confirmed the proapoptotic properties of ZnO NPs. Notably, Alami et al. (2020) and Mahdizadeh et al. (2019) have reported that ZnO NPs induce apoptosis in tumor cells by upregulating caspases [44, 45] Moreover, our study revealed that ZnO NPs enhance caspase expression and induce DNA damage, consistent with the findings of Mahdizadeh et al. (2019) [45].

Additionally, our study′s essential findings demonstrated that the presence of ZnO NPs leads to a dose‐dependent decrease in Bcl‐2 expression and an increase in BAX expression. The Bcl‐2 family proteins play a role in outer mitochondrial membrane permeabilization and the modulation of mitochondrial homeostasis, thereby influencing cell fate, including the loss of mitochondrial membrane potential (MMP) [46, 47]. Scientists have established the interplay between proapoptotic and antiapoptotic members of the Bcl‐2 family as a regulatory circuitry that controls cell fate. Additionally, oxidative stress, particularly active superoxide, can trigger the intrinsic apoptotic pathway by causing mitochondrial membrane permeabilization [48, 49]. Various studies have indicated that an increased Bax/Bcl‐2 ratio activates caspase 3, promoting apoptosis. However, conflicting reports suggest that Bax translocation can regulate caspase three and induce apoptosis, even without altering the Bax/Bcl‐2 ratio [50, 51].

Moreover, our study revealed that ZnO NPs downregulate the expression of two genes, PKM2 and Hk2, in HT‐29 cells. These findings are consistent with the results reported by Lai et al. [43]

## 5. Conclusion

The present study demonstrates the environmentally friendly synthesis of ZnO NPs using *Lactobacillus plantarum* bacteria. Moreover, the study shows that ZnO NPs can induce apoptosis by affecting DNA and the expression of genes associated with cell division. Additionally, they inhibit cell synthesis and division in HT‐29 cells. Based on the results of this study and other research on the antitumor properties of ZnO NPs, it is reasonable to consider their application in biomedical settings and the development of anticancer drugs.

## Author Contributions

Mohammadreza Azimi and Anoosh Eghdami performed the experiments, conceived and designed the study, and wrote, analyzed, funded, and critically revised the manuscript. Bahram Keyvani, Ramin Cheraghali also actively helped in the experiments and participated in the study design, study implementation, and manuscript revision.

## Funding

No funding was received for this manuscript.

## Ethics Statement

The authors have nothing to report.

## Consent

The authors provide consent to publish.

## Conflicts of Interest

The authors declare no conflicts of interest.

## Data Availability

The datasets generated during and/or analyzed during the current study are available from the corresponding author on reasonable request.
